# Revisiting FR13A for submergence tolerance: beyond the *SUB1A* gene

**DOI:** 10.1093/jxb/erae299

**Published:** 2024-07-12

**Authors:** Waseem Hussain, Mahender Anumalla, Abdelbagi M Ismail, Harkamal Walia, Vikas Kumar Singh, Ajay Kohli, Sankalp Bhosale, Hans Bhardwaj

**Affiliations:** International Rice Research Institute (IRRI), Los Baños, Laguna 4031, Philippines; International Rice Research Institute (IRRI), Los Baños, Laguna 4031, Philippines; IRRI-South Asia Hub (IRRI-SA), Hyderabad, Telangana, 502324, India; International Rice Research Institute (IRRI), Los Baños, Laguna 4031, Philippines; Department of Agronomy and Horticulture, University of Nebraska-Lincoln, Lincoln, NE 68583, USA; International Rice Research Institute (IRRI), Los Baños, Laguna 4031, Philippines; IRRI-South Asia Regional Centre (IRRI-SARC), Varanasi, Uttar Pradesh, 221106, India; International Rice Research Institute (IRRI), Los Baños, Laguna 4031, Philippines; International Rice Research Institute (IRRI), Los Baños, Laguna 4031, Philippines; International Rice Research Institute (IRRI), Los Baños, Laguna 4031, Philippines; Hong Kong Baptist University

**Keywords:** Connected breeding, FR13A, recovery ability, rice, *SUB1A*, submergence

**Rice landrace FR13A is the original donor of the *SUB1*A gene. FR13A harbors hidden genetic variation that surpasses the submergence tolerance level of *SUB1A*. Here, we provide an overview of the unique features of FR13A associated with exceptional recovery ability that have been overlooked since the significant discovery of the *SUB1* locus. We present a detailed overview of how to underpin the new genes associated with the recovery ability seen in FR13A. Finally, we propose a unique approach to develop rice cultivars that surpass submergence tolerance beyond the *SUB1* locus from FR13A.**

Submergence stress results from the complete inundation of rice plants when flash floods occur during the vegetative or seedling stage, and can last up to 2 weeks. Submerged plants encounter an energy crisis, which may result in whole-plant mortality and hence grain yield is reduced (Yuan *et al.*, 2023). In recent years, submergence has become more frequent and sometimes extends for >20 d in rainfed areas, possibly due to climate change (Shu *et al.*, 2023). FR13A, an Indian flood‐tolerant variety derived from a traditional landrace, Dhalputtia, through single seed selection, can thrive in flash floods for 2 weeks or more (Xu and Mackill, 1996; Xu *et al.*, 2006). Several other landraces showed a high tolerance for flash flooding, which has not been sufficiently exploited in breeding (Ismail and Mackill, 2014). However, among all landraces, FR13A has been recognized as one of the most flash flood-tolerant genotypes (Ismail and Mackill, 2014; Singh *et al.*, 2017).

A major scientific breakthrough identified a submergence tolerance *SUB1* quantitative trait locus (QTL) on chromosome 9 in FR13A (Xu and Mackill, 1996). The *SUB1* locus contains three ethylene-responsive factor (ERF) genes, namely *SUB1A*, *SUB1B*, and *SUB1C* (Xu *et al.*, 2006). Subsequently, *SUB1A* was confirmed as the causal gene providing submergence tolerance at the *SUB1* locus. Plants with the *SUB1A* gene employ a unique quiescence strategy to manage submergence (Bailey-Serres *et al.*, 2010). They suppress ethylene-activated leaf or shoot elongation, thereby reducing carbohydrate consumption. This strategy allows them to conserve energy, ensuring survival even in submergence conditions. Upon the water receding, the plants with *SUB1A* recover rapidly and continue growing. On the other hand, plants without the *SUB1A* gene undergo rapid leaf and internode elongation when submerged, leading to fast depletion of energy and death if submergence is prolonged.

Remarkable success has been achieved in introducing *SUB1A* into several mega rice varieties, making them tolerant to flash floods for up to 2 weeks (Ismail *et al.*, 2013). However, under prolonged submergence, probably exacerbated by climate change (Shu *et al.*, 2023), the *SUB1A* gene alone cannot provide complete submergence tolerance for more extended periods and under fluctuating flooding conditions (Singh *et al.*, 2017; Anumalla *et al.*, 2023). Supplementing *SUB1A* with additional tolerant genes for developing the next-generation rice varieties with enhanced submergence tolerance for prolonged flooding is urgently needed. New genes for submergence tolerance have been reported in rice (Ismail, 2018). However, these additional genes have not been successfully leveraged to boost submergence tolerance in rice (Gonzaga *et al.*, 2017).

Submergence tolerance is a multiphasic trait associated with underwater survival and recovery ability after flood waters recede (Yeung *et al.*, 2019). Recovery ability refers to how surviving plants exhibit faster regrowth, generate new tillers and leaves more rapidly, and accumulate greater biomass during post-submergence recovery. A plant’s survival after submergence depends on its ability to repair damage, reoxygenate, and reactivate essential processes during post-submergence recovery (Yeung *et al.*, 2019). Survival score changes have been linked to recovery ability, which may result from various genetic events occurring pre- and/or post-submergence (Singh *et al.*, 2014; Yeung *et al.*, 2019). Also, post-submergence growth features associated with faster regrowth positively correlate with survival scores (Sarkar and Bhattacharjee, 2011; Singh *et al.*, 2014, 2017). Notably, genotypes with higher survival rates tend to recover more quickly.

Various past efforts dissected the additional genetics of FR13A and revealed new QTLs other than *SUB1* for submergence tolerance on chromosomes 1, 2, 5, 7, 8, 10, and 11 (Sripongpangkul *et al.*, 2000; Gonzaga *et al.*, 2016, 2017). However, all the past studies focused on using the survival score as a main phenotypic trait for mapping additional submergence QTLs. We believe additional genetic variation in FR13A is associated with the recovery ability rather than the survival percentage. No concrete efforts have been made to dissect the genetics of the recovery ability in FR13A in the mapping populations derived by crossing with FR13A. Further, all the studies except that by Gonzaga *et al.* (2017) focused on identifying the submergence QTLs using the mapping populations where *SUB1* was segregating with non-*SUB1* QTLs, masking and preventing the accurate estimate of non-*SUB1* QTLs. The limited success in identifying the reliable additional QTLs beyond the *SUB1* locus from FR13A is due to the fact that the target phenotype and mapping populations leveraged were not suitable for dissecting the recovery ability of FR13A.

In this Viewpoint, we discuss what makes FR13A uniquely resilient to prolonged flash flooding. We present evidence for untapped variation in FR13A that enhances its submergence tolerance beyond the *SUB1A* locus. We showed that the better and faster recovery ability of FR13A is associated with the improved submergence tolerance of FR13A. We present an approach for tapping this additional genetic variation of FR13A to reveal additional genes and understand submergence tolerance better. Instead of continued exploration of wider germplasm to discover new genes for submergence tolerance, we suggest revisiting FR13A. We provide a perspective on how we can dissect the additional genetics in FR13A and identify additional submergence genes. What phenotypic setup and submergence screening duration can enable this genetic dissection and mapping analysis? How do we utilize FR13A to breed and develop better submergence-resilient rice varieties when additional genes for submergence tolerance are unknown?

## Why revisit FR13A: is it more than *SUB1A?*

Among all the known landraces for submergence tolerance, FR13A stands out as one of the most resilient genotypes for flash floods (Singh *et al.*, 2017). We chose FR13A for our study because of its extraordinary ability to survive submergence beyond 2 weeks. In a highly replicated study (3 years with two seasons each year) with submergence lasting up to 3 weeks, we observed that the survival rate of the *SUB1A* genotypes did not exceed 40% (Box 1). On the other hand, FR13A, a donor for *SUB1A*, had a survival rate of >90% in all the experiments. Interestingly, the *SUB1A* introgression genotypes revealed significant genotype×environment (G×E) interactions, changing their survival ranking across the environments (Box 1).

Box 1. **Distinctive features of FR13A associated with survival growth, recovery ability, and stability**

(A) Survival percentage of the FR13A donor for the *SUB1A g*ene, non-*SUB1A* genotypes, and genotypes introgressed with the *SUB1A* gene under 21 d of submergence. FR13A shows a high rate of survival as compared with *SUB1A* introgression lines. The error bar indicates the mean ±SE. (B) Recovery shown as the difference in survival rate over time of FR13A and *SUB1A* introgression lines after de-submergence. The number of seedlings that survived and recovered was recorded at three time points: 7, 14, and 21 d after the de-submergence to check the recovery rate, highlighted in different colors. FR13A shows the same survival percentage at all three time points. However, *SUB1A* introgressed genotypes showed a significant drop in the survival rate at 14 d and 21 d, indicating the slower recovery rate of *SUB1A* introgressed genotypes. The * shows the significance level at *P*<0.05; ns means non-significant with *P*>0.05. The differences in survival rate between 14 d and 21 d were non-significant. (C) The G×E interactions of survival percentage for FR13A and *SUB1A* introgression genotypes. A strong cross-over interaction with *SUB1A* genotypes indicates strong G×E interactions and high unpredictability across the environments under 21 d of submergence screening. The *P*-value and significance for G×E are given at the end of the line for each genotype.

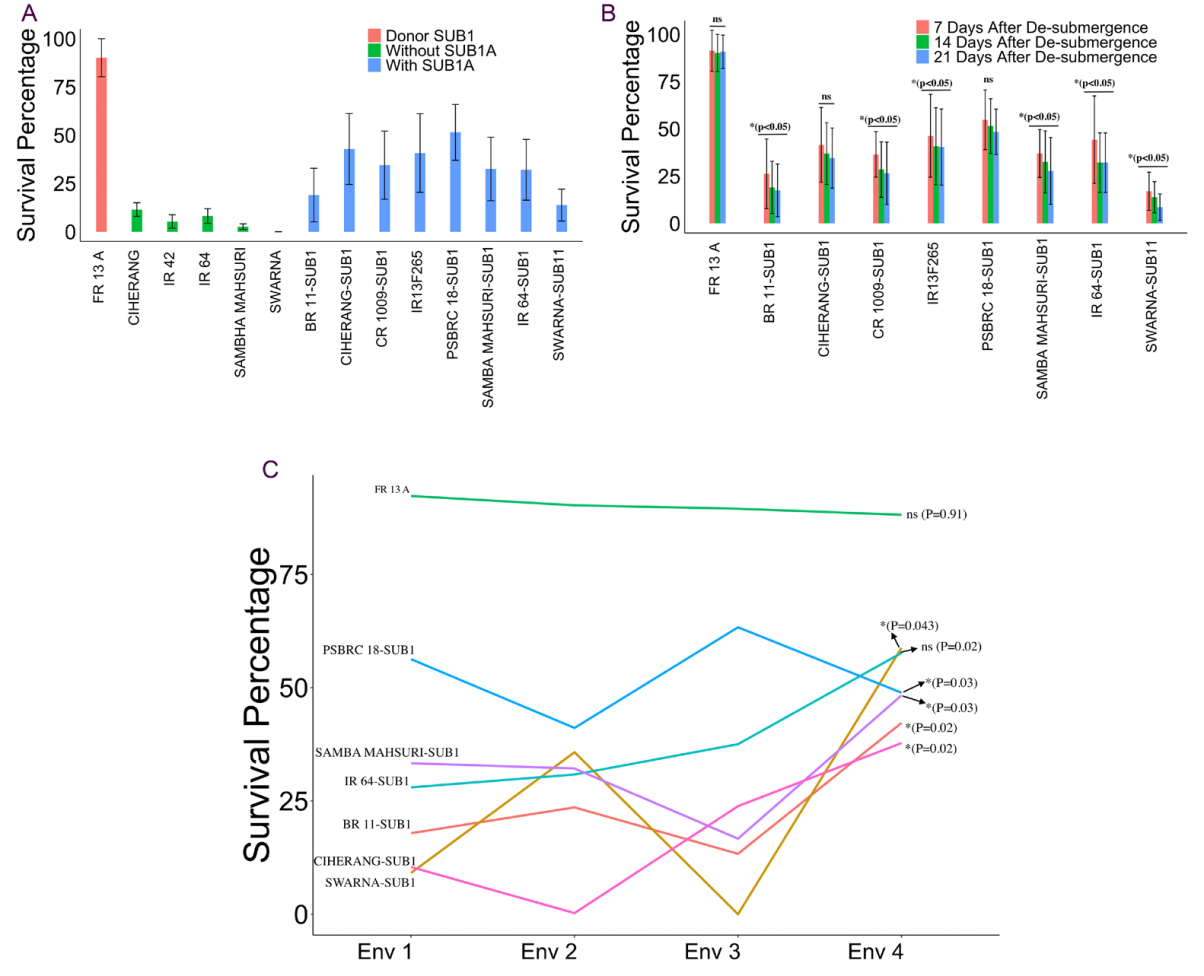

On the other hand, genotype FR13A showed non-significant G×E interactions and was highly stable across the environments. These replicated field experiments indicate that under prolonged submergence of 2 weeks or more, the *SUB1A* gene is highly unstable (Box 1), and the survival rate is significantly reduced (Sarkar and Bhattacharjee, 2011; Gonzaga *et al.*, 2016, 2017; Singh *et al.*, 2017). It is also well established in the literature that the *SUB1A* introgression lines do not add up to the tolerance level of FR13A (Singh *et al.*, 2014, 2017; Anumalla *et al.*, 2023). It was shown that FR13A not only survived 3 weeks of complete submergence but also exhibited remarkable plant height, elongation ability, and rapid regrowth during re-emergence, setting it apart from *SUB1* introgression lines (Sarkar and Bhattacharjee, 2011). Thus, it is clear that FR13A is not just a *SUB1A* locus genetic variation; it is much more, and a significant genetic variation lies hidden in FR13A. The high survival percentage, even under 3 weeks or more of submergence, and the huge stability due to the absence of G×E interactions make FR13A unique and the most desired genetic resource for revisiting.

## What is missing in *SUB1A* introgression genotypes and present in FR13A?

The better and faster recovery ability of FR13A is associated with its enhanced submergence tolerance. This study evaluated recovery ability by comparing survival rates at 7, 14, and 21 d after de-submergence. Significant differences in survival scores across these time points revealed variations in regrowth ability among the *SUB1A* genotypes and FR13A (Box 1). Additionally, we assessed the recovery ability by visually observing the strong green stem, broad green leaves, and taller shoot in FR13A compared with *SUB1A* introgression genotypes (Box 2). We argue that these traits are distinctive phenotypic features present in FR13A and are associated with the higher survival percentage and quicker regrowth of FR13A (Box 2). The regrowth ability of the *SUB1A* introgression genotypes was moderate and, under prolonged submergence, recovery ability akin to that of FR13A was missing in *SUB1A* introgression lines (Boxes 1, 2). The recovery ability is potentially a new target trait to genetically dissect and will underpin the new genes for enhanced submergence tolerance. Previous research has provided valuable insights and gained a better understanding of the physiological mechanisms of the recovery ability of FR13A following de-submergence (Singh *et al.*, 2017; Yeung *et al.*, 2019; Yuan *et al.*, 2023). However, the genetic dissection and finding the new genes or QTLs underlying FR13A’s exceptional recovery ability have been somewhat neglected, particularly since the discovery of the *SUB1* locus.

Box 2. **Extraordinary recovery ability of FR13A under different situations**

(A) FR13A after 6 weeks of de-submergence at the IRRI-HQ field, where the *SUB1A* lines showed little to no survival. The field was submerged for 3 weeks. (B) FR13A during day one of de-submergence. FR13A has a solid green stem and leaves, and stands as an erect plant compared with *SUB1A* introgression lines. The SUB1A introgression genotype is lying flat in the field and will start recovering slowly. (C) A direct comparison of FR13A with two *SUB1*A introgression genotypes grown in pots submerged for 3 weeks in the field. FR13A has a solid and erect stem and green leaves compared with *SUB1A* genotypes.

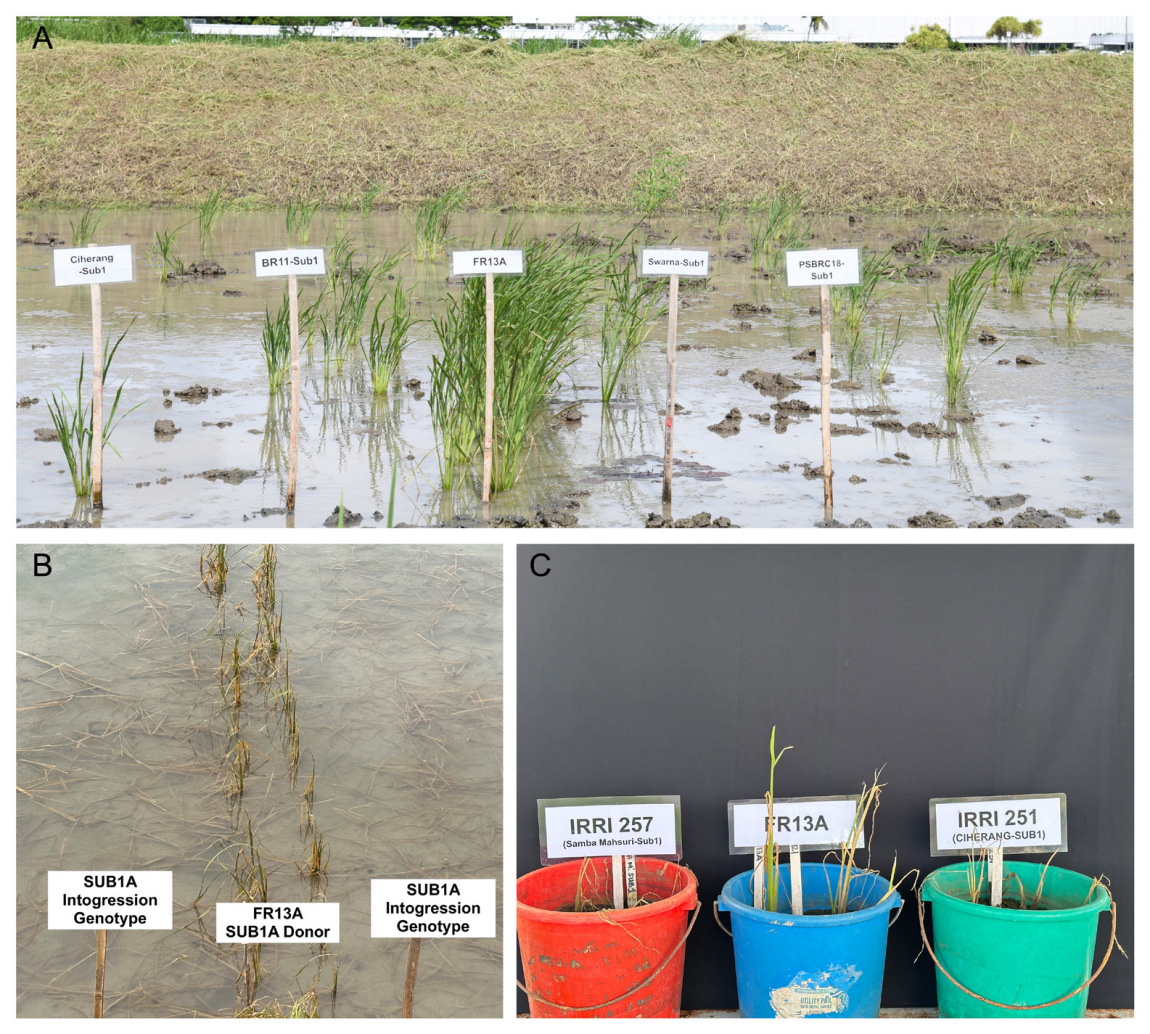

## What next? Shift of focus on the genetic dissection of recovery ability

The extraordinary recovery capacity of FR13A makes it unique for better resilience to prolonged flash flooding, extending well beyond 2 weeks. Previous research has primarily emphasized survival rates or related traits rather than the recovery aspect when searching for the additional submergence tolerance genes in FR13A (Sripongpangkul *et al.*, 2000; Gonzaga *et al.*, 2016, 2017). Shifting the focus to genetically dissecting FR13A’s recovery ability may uncover novel genes and a better understanding of submergence tolerance. To mainly focus on the recovery ability and genetically dissect it, we suggest creating a unique mapping population derived by crossing FR13A with *SUB1A* introgression lines. In this type of mapping population, the genetic variation will not segregate within the *SUB1A* locus, which is wholly fixed, and any additional genetic variation emerging in this population would be from different loci or genetic mechanisms. We suggest screening the mapping population at three levels of submergence screening: 1 week, 2 weeks, and 3 weeks after de-submergence. Screening the derived mapping populations for 3 weeks or more underwater is key to obtaining a striking difference and a reliable phenotype of survival percentage and recovery ability for genetic analysis.

The key is to identify the target traits associated with recovery ability and use them for genetic association analysis. Our early research suggests measuring morphological features of the recovery ability using the presence and absence of a firm and green stem, biomass percentage, and stem and leaf elongation as primary phenotypic traits (Box 2). Besides phenotypic characteristics, we suggest characterizing the mapping population’s complete physiology and metabolome to understand the faster recovery of FR13A (Yeung *et al.*, 2019). At the physiological and metabolome levels, more traits, chlorophyllase enzyme activity, chlorophyll content, photosynthetic capacity, carbohydrate reserves in the stem, and metabolites can be associated with better recovery (Voesenek and Bailey-Serres, 2013; Locke *et al.*, 2018; Lin *et al.*, 2024).

Further, studying the FR13A×*SUB1A* mapping populations at multi-omics levels (genome, transcriptome, proteome, and metabolome) integrated with high-throughput phenotyping at different levels of submergence screening is worthwhile to solve the hidden mysteries of the recovery ability and to gain a better understanding of plant stress resilience during recovery (Yeung *et al.*, 2019; Yuan *et al.*, 2023). We also emphasize a major paradigm shift in studying the mechanisms of recovery ability by comparing the *SUB1A* introgression lines directly with FR13A rather than a comparison of *SUB1A* introgression lines with the non-*SUB1* genotypes to avoid masking the effects of the *SUB1* locus variation. The power of genome editing can be harnessed to knock out the *SUB1A* locus (Barrero *et al.*, 2022) from FR13A and derived mapping populations and study the response of genome-edited genotypes in depth at the phenotypic, physiological, and molecular levels after de-submergence.

## Enhancing submergence tolerance beyond *SUB1A*

As the additional genes and their genetic architecture are unknown in FR13A, we propose our newly developed connected breeding approach (Khanna *et al.*, 2024) to introduce the additional genetic variation of FR13A into the popular rice varieties or elite pool and boost the *SUB1A* gene tolerance level. The connected breeding approach (more details given in Box 3) uses the bridge breeding scheme (Sanchez *et al.*, 2023) to enrich the elite gene pool with additional complex genetic variation (Box 3). In the bridge scheme, bridge materials are generated by crossing a donor with an elite (DE), which is then used to cross with the elite pool genotypes rather than a direct crossing of a donor with the elite genotype. This bridge scheme is used primarily to avoid gene pool contamination with undesirable alleles and pushing back the mean performance of the elite population, which has been improved for generations (Longin and Reif, 2014). In the past, and before the discovery of the *SUB1A* gene, FR13A was used as a parent by directly crossing it with a popular rice variety to introduce submergence tolerance. However, the approach of direct crossing through the conventional approach was unsuccessful as it failed to de-couple the undesirable agronomic traits and the submergence tolerance trait in the identified derived progenies of the crosses (Emerick and Ronald, 2019). Thus, our newly developed connected breeding approach, leveraging the bridge materials in association with genomic selections, speeding up breeding, and phenotyping, offers tremendous potential to overcome the limitations of linkage drag (Toulotte *et al.*, 2022) and systematically incorporate the complex genetic variation in the elite pool. This approach provides the great benefit of combining a desirable genetic variation in the genetic background of popular varieties when the genes are unknown and genetic architecture is complex.

Box 3. **Connected breeding approach to introduce the complex genetic variation from a donor into the elite pool**

(A) The donor, here FR13A, has a novel genetic variation, and the genetic markers or genomic regions for the trait are unknown. (B) The approach to generate the bridge materials (DE) by crossing the most recent best elite line with high breeding value for grain yield from the ongoing breeding program (shown in C) with a donor. Speed breeding, phenotyping (here 21 d of submergence screening), and genomic selection using a relationship matrix based on markers are leveraged to select the best genotype as the connected genotype (DE) having trait variation from the donor and high breeding value from the elite genotype. Different circles and arrows indicate that multiple rounds of the breeding cycle can be performed to overcome linkage drag and avoid the undesirable agronomic features associated with the donor background, which is a unique feature of connected breeding. The breeding cycle can be accelerated to within one and half years using the speed breeding facility. (C) Once the best genotype (DE) is identified, it can be crossed with other high-yielding elite lines of the breeding program to generate better high-yielding elite lines with stronger submergence tolerance for further field assessment. The whole process can be repeated at every breeding cycle with different donors, keeping the donor-derived bridge materials connected to the ongoing breeding scheme; that is why we call it connected breeding. Every breeding cycle, a new best elite genotype will be selected from the elite breeding and crossed with another donor to develop new DE genotypes to ensure sustainable genetic gains and maintain the high diversity in the elite pool.

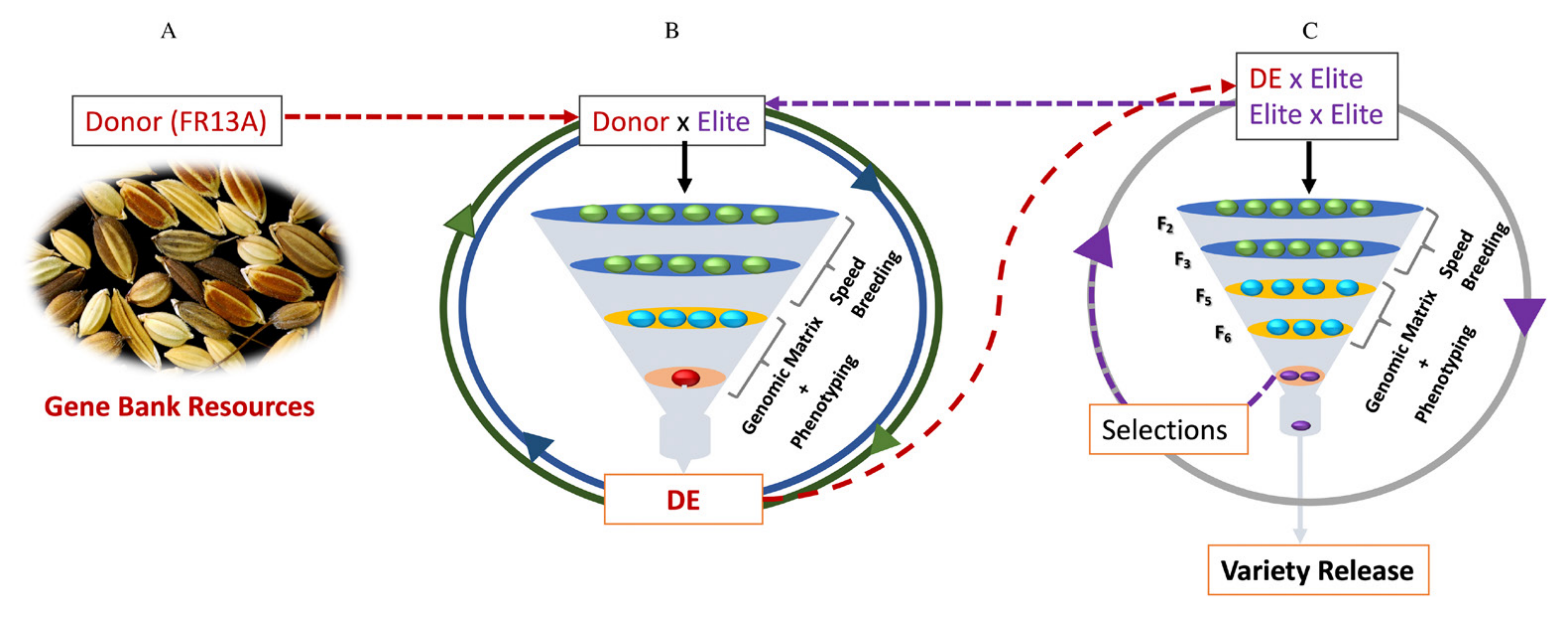

## Outlook

FR13A, a donor for the *SUB1A* gene, harbors additional genetic variation for submergence tolerance and is the best germplasm resource worth exploring further. We provide evidence that *SUB1A* introgression lines do not add up to the tolerance level of FR13A. The genetic variation in FR13A for submergence tolerance is far beyond *SUB1A*, and significant hidden genetic variation is present in FR13A, mainly associated with its recovery ability. The recovery ability of FR13A should be the next target for exploring and identifying new genes for enhanced submergence tolerance. The mapping population of FR13A×*SUB1A* in three diverse backgrounds initiated at the IRRI will serve as an excellent genetic resource for the global rice research community to dissect the hidden genetics of FR13A and identify new genes to boost submergence tolerance in rice. We have made a strong case for exploring, at depth, the underlying physiology and genetics of FR13A to identify additional genes associated with prolonged submergence tolerance by combining phenotyping of superior recovery traits with a multi-omics approach and genome editing. This, in turn, is expected to improve the submergence tolerance beyond what has already been achieved by incorporating the *SUB1A* gene alone.
