# Correction to: Revisiting FR13A for submergence tolerance: beyond the *SUB1A* gene

**DOI:** 10.1093/jxb/erae397

**Published:** 2024-09-26

This is a correction to: Waseem Hussain, Mahender Anumalla, Abdelbagi M Ismail, Harkamal Walia, Vikas Kumar Singh, Ajay Kohli, Sankalp Bhosale, Hans Bhardwaj, Revisiting FR13A for submergence tolerance: beyond the *SUB1A* gene, *Journal of Experimental Botany*, 2024; https://doi.org/10.1093/jxb/erae299

In the originally published version of the manuscript, there was a typographical error in the first name of the fourth author. This has been emended to read: “Harkamal Walia”.

